# Tuning of AcurosXB source size setting for small intracranial targets

**DOI:** 10.1002/acm2.12091

**Published:** 2017-05-04

**Authors:** Stephen J. Gardner, Siming Lu, Chang Liu, Ning Wen, Indrin J. Chetty

**Affiliations:** ^1^ Department of Radiation Oncology Henry Ford Health System Detroit MI USA; ^2^ Department of Medical Physics Memorial Sloan Kettering Cancer Center Commack NY USA

**Keywords:** radiochromic film dosimetry, small field dosimetry, SRS dose delivery

## Abstract

This study details a method to evaluate the source size selection for small field intracranial stereotactic radiosurgery (SRS) deliveries in Eclipse treatment planning system (TPS) for AcurosXB dose calculation algorithm. Our method uses end‐to‐end dosimetric data to evaluate a total of five source size selections (0.50 mm, 0.75 mm, 1.00 mm, 1.25 mm, and 1.50 mm). The dosimetric leaf gap (DLG) was varied in this analysis (three DLG values were tested for each scenario). We also tested two MLC leaf designs (standard and high‐definition MLC) and two delivery types for intracranial SRS (volumetric modulated arc therapy [VMAT] and dynamic conformal arc [DCA]). Thus, a total of 10 VMAT plans and 10 DCA plans were tested for each machine type (TrueBeam [standard MLC] and Edge [high‐definition MLC]). Each plan was mapped to a solid water phantom and dose was calculated with each iteration of source size and DLG value (15 total dose calculations for each plan). To measure the dose, Gafchromic film was placed in the coronal plane of the solid water phantom at isocenter. The phantom was localized via on‐board CBCT and the plans were delivered at planned gantry, collimator, and couch angles. The planned and measured film dose was compared using Gamma (3.0%, 0.3 mm) criteria. The vendor‐recommended 1.00 mm source size was suitable for TrueBeam planning (both VMAT and DCA planning) and Edge DCA planning. However, for Edge VMAT planning, the 0.50 mm source size yielded the highest passing rates. The difference in dose calculation among the source size variations manifested primarily in two regions of the dose calculation: (1) the shoulder of the high‐dose region, and (2) for small targets (volume ≤ 0.30 cc), in the central portion of the high‐dose region. Selection of a larger than optimal source size can result in increased blurring of the shoulder for all target volume sizes tested, and can result in central axis dose discrepancies in excess of 10% for target volumes sizes ≤ 0.30 cc. Our results indicate a need for evaluation of the source size when AcurosXB is used to model intracranial SRS delivery, and our methods represent a feasible process for many clinics to perform tuning of the AcurosXB source size parameter.

## 1 Introduction

Stereotactic radiosurgery (SRS) has become a valuable treatment modality to treat lesions within the brain^1^ and spine.^2^ In particular, SRS provides a non‐invasive treatment approach for unresectable tumors (such as those in the eloquent cortex or otherwise deep‐seated tumors) or for patients who are otherwise not candidates for surgery.^3^

Though SRS was first performed using a specialized device, now commercially available as the GammaKnife, technological advances have allowed for stereotactic therapies using the linear accelerator. Several noteworthy advances have allowed for improved precision in linear accelerator‐based SRS: (1) the advent of treatment room stereotactic imaging systems,^4^, ^5^, ^6^, ^7^ including stereoscopic planar imaging and cone‐beam CT imaging, (2) improvements in patient support devices, including 6 degree‐of‐freedom capabilities in the treatment couch^8^ and improvements in couch movement precision, (3) increasing availability of high‐intensity photon modes,^9^, ^10^, ^11^ such as flattening‐filter free photon modes with dose rates up to 2400 MU/min, (4) high‐definition multi‐leaf collimator (MLC) systems with leaf widths as narrow as 2.5 mm,^12^, ^13^ and (5) optical monitoring systems to track patient motion throughout the treatment course.^14^, ^15^

Of course, the advances in the preceding paragraph all focus on the treatment delivery, while an accurate end‐to‐end treatment delivery relies on the marriage of the dose modeling within the treatment planning system and the physical dose delivery within the treatment room. Along these lines, the modeling of small‐field dose delivery has garnered much interest. The accurate measurement and modeling of small‐field dose delivery (i.e., field sizes < 3 × 3 cm^2^ in water‐equivalent media) has many challenges, including the effects of the finite size of the radiation source, loss of charged particle equilibrium (CPE), and sensitivity to small changes in field size for perturbation factors of ion chambers used for measurement.^16^ The AcurosXB algorithm gives a discretized solution to the linear Boltzmann transport equation,^17^, ^18^, ^19^ which provides improvements in regions with loss of CPE, such as heterogeneity interfaces.^20^, ^21^ However, the proper selection of source size within the AcurosXB algorithm is still essential for the accurate modeling of small field deliveries. The purpose of this study was two‐fold: (1) to present a clinically achievable method to evaluate the source size for small‐field dose calculation, and (2) to use the method to evaluate the ideal source size setting within AcurosXB for flattening‐filter energy modes for two delivery platforms (Varian Edge and TrueBeam machines), MLC leaf models (Millennium120 HD‐MLC and standard Millennium120 MLC), and delivery techniques for intra‐cranial SRS planning (DCA and VMAT).

## 2 Methods

### 2.A Treatment planning and beam modeling

A total of 40 cranial SRS plans were generated for analysis in this IRB‐approved retrospective study. The analysis included two machine types with different MLC models: (1) Varian Edge machine (SN: 2003), which was installed in 2014 with Millennium120 HD‐MLC (central bank MLC leaf width 0.25 cm), and (2) Varian TrueBeam machine (SN: 2440), which was installed in late 2015 with standard Millennium120 MLC (central bank MLC width 0.5 cm). For each machine, plans were generated for two delivery modalities (dynamic conformal arc [DCA] and volumetric modulated arc therapy [VMAT]). Thus, for each machine type, the analysis included 10 DCA plans and 10 VMAT plans. The two different delivery modalities were selected to highlight potential differences between intensity‐modulated deliveries (VMAT) and deliveries similar to MLC‐defined open fields (DCA).

All planning and dose calculations were performed using Varian Eclipse (v. 13) TPS and AcurosXB dose calculation algorithm (v. 13; 1.0 mm isotropic dose calculation grid size and dose to medium calculation setting). The dose to medium setting was used in accordance with recently published recommendations from the NRG physics group.^22^ The current study was designed to evaluate the source size setting for small intracranial targets, with target volumes as small as 0.03 cc (which corresponds to approximately 4 mm diameter); in addition, several other studies have used smaller dose grid size (i.e., less than 1.5 mm) as the standard for dose calculation comparisons when evaluating small‐field dosimetry.^23^, ^24^, ^25^, ^26^ With this in mind, this study used 1.0 mm dose grid size for all dose calculations. All planning was done with 6x‐FFF beam energy with nominal dose rate set to the maximum setting (1400 MU/min). The patient treatment plan was generated with beam model parameters following the vendor recommendations for source size (spot size setting of 1.00 mm in X‐ and Y‐directions) and our current clinical values for MLC parameters (i.e., dosimetric leaf gap (DLG) and MLC leaf transmission value).^7^ The details of the relevant treatment planning data (including target volume size and location) are shown in Table 1 for Edge linac and Table 2 for TrueBeam linac.

**Table 1:** Relevant treatment planning data for (a) DCA and (b) VMAT planning for Edge linac

Patient number	Disease site	PTV volume (cc)	Rx dose (Gy)	Max film plane dose (Gy)
(a) Varian edge – DCA planning				
1	Lt frontal	0.04	18	13.67
2	Rt ant frontal	0.07	18	12.65
3	Lt frontal	0.03	20	14.7
4	Lt frontal	0.23	18	15.69
5	Rt parietal	0.04	18	14.65
6	Lt inf frontal	1.29	18	14.49
7	Lt temporal	0.33	18	13.01
8	Cerebellar	0.48	18	18.55
9	Rt frontal	0.32	18	14.48
10	Rt parietal	0.19	18	14.45
(b) Varian edge – VMAT planning				
1	Rt parietal	0.31	20	14.47
2	Rt ant frontal	0.07	18	16.63
3	Rt temporal	0.39	18	16.23
4	Rt CPA	0.86	16	16.43
5	Lt frontal	0.03	20	17.92
6	Lt frontal	0.23	18	16.16
7	Lt temporal	1.29	18	15.6
8	Lt inf frontal	0.33	18	17.21
9	Rt acoustic	0.75	13	12.36
10	Lt brainstem	0.51	9	10.64

**Table 2:** Relevant treatment planning data for (a) DCA and (b) VMAT planning for TrueBeam linac

Patient number	Disease site	PTV volume (cc)	Rx dose (Gy)	Max film plane dose (Gy)
(a) Varian truebeam – DCA planning				
1	Rt temporal	2.01	18	14.66
2	Rt parietal	0.85	18	12.17
3	Rt frontal	0.82	18	12.07
4	Lt cerebeller	1.01	18	15.12
5	Rt parietal	1.93	18	14.13
6	Rt cerebeller	1.64	18	15.9
7	Rt thalamus	0.69	18	17.65
8	Lt frontal	0.46	18	13.78
9	Lt cerebeller	0.49	18	19.97
10	Lt frontal	0.38	18	12.03
(b) Varian truebeam – VMAT planning				
1	Lt parietal	0.67	18	15.96
2	Rt precentral	0.78	18	15.88
3	Resection cavity SRS	13.9	15	13.99
4	Rt acoustic neuroma	0.43	13	14.13
5	Rt parietal	1.22	18	16.97
6	Lt temporal	0.24	18	16.84
7	Lt inf frontal	0.33	18	18.29
8	Lt temporal	1.29	18	14.94
9	Lt acoustic	1.16	12	11.34
10	Rt frontal	2.07	18	16.62

To study the influence of source size on the calculated dose for cranial SRS deliveries, a total of 5 AcurosXB beam models were created for each machine type. The user can tune the source size in the beam configuration module through varying the effective target spot size value, which is entered by the user separately for X‐ and Y‐directions.^19^ All beam models used the same input measured data (percent depth‐dose, cross‐line profiles, and output factors), with the source size varied from 0.50 mm to 1.50 mm in 0.25 mm increments for each machine type. Each beam model was then calculated separately with its unique source size value. In addition to the source size parameter, the DLG was also varied in the analysis. In Eclipse, the DLG represents the TPS method for modeling of the rounded MLC leaf end.^19^ For small MLC‐defined fields, the leaf end modeling and the potentially partial viewing of the finite size of the radiation source along the central axis are inherently coupled. The DLG parameter is not included within the Beam Configuration workspace in ARIA v. 13; rather, it is included in the machine properties of the RT Administration workspace. Nonetheless, the DLG parameter was also varied in our analysis: three DLG values were included in the modeling and calculation analysis for each machine type.

All treatment plans were mapped to a water‐equivalent slab phantom, with total phantom dimensions of 15 cm × 30 cm × 30 cm (Gammex Inc., Middleton, WI, USA). The isocenter was placed in the center of the phantom, corresponding to 7.5 cm depth. Each treatment plan was calculated for all combinations of source size beam model and DLG value. Thus, for each treatment plan, a total of 15 dose calculations were performed to sample the various source size and DLG values for dose calculation. All dose calculations were performed with the same monitor unit values determined during the original plan optimization. After dose calculation was completed, planar dose planes were exported for analysis (512 × 512 matrix resolution, 5 cm × 5 cm matrix size).

### 2.B. Gafchromic film measurements and calibration

Film measurements using Gafchromic EBT3 film (Film Size: 20.3 × 25.4 cm^2^; Ashland Inc., Covington, KY, USA) were used to evaluate the dose calculation accuracy in this study. Gafchromic film was selected due to several attractive detector properties: extremely high spatial resolution, large planar detection area, minimal directional dependence, and low energy dependence. In addition, Gafchromic film and associated dosimetric analysis tools are widely available to the radiation oncology community, making our methods described here feasible for many clinics. The films were handled according to the recommendations of AAPM Task Group 55.^27^ The phantom localization and treatment procedure followed our clinical process for intracranial SRS treatment delivery. Specifically, the phantom and film plane (coronal plane at mid‐phantom – 7.5 cm depth) were localized using CBCT imaging prior to dose delivery (125 kVp, Full‐fan filter, 1 mm slice thickness), and all plans were delivered at planned gantry, collimator, and couch angles. The average delay time between irradiation and film scanning was approximately 24 hr. Films were scanned in an Epson Expression 10000XL flatbed scanner (Seiko Epson Corp, Nagano, Japan). All films were scanned at the center of the scanner bed with resolution settings of 150 dot per inch and 48 bit RGB mode (16 bits per color channel). A four‐way flip method was used to average out any intrinsic light source non‐uniformity of the scanner. Dosimetric analysis was done via green channel due to its superior sensitivity at the dose levels larger than 10 Gy.^28^

The film calibration and dosimetric analysis was performed using in‐house software. Calibration films were irradiated in a nine square dose pattern (area of 2 × 2 cm^2^ per square). The in‐house calibration routine matches the film optical densities within each square to the TPS calculated dose for the same beam geometry. Then, a calibration curve was generated using cubic polynomial least squares fitting.

### 2.C. Dose distribution analysis

**Figure 1:**
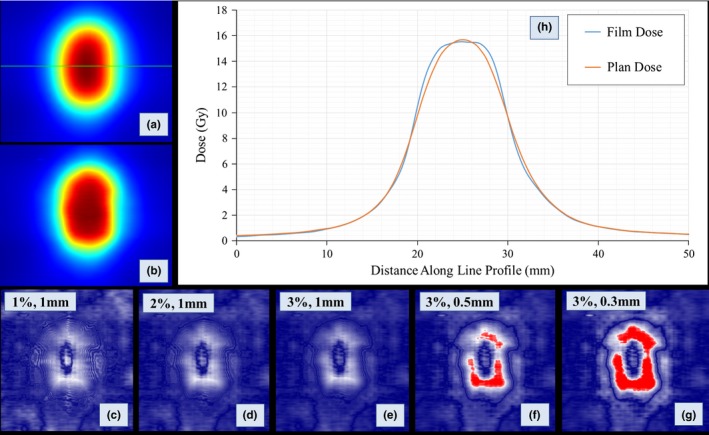
Comparison of Gamma analysis criteria for one representative case from the study. The red regions in each Gamma map represent failing pixels for the relevant Gamma criteria used. a, Planned dose (1.50 mm source size) plane with line profile geometry (green horizontal line). b, Green channel film dose plane. c, Gamma map for 1%,1 mm criteria. d, Gamma map for 2%, 1 mm criteria. e, Gamma map for 3%, 1 mm criteria. f, Gamma map for 3%, 0.5 mm criteria. g, Gamma map for 3%, 0.3 mm criteria. h, Line profile comparing the AcurosXB planned dose (1.50 mm source size) and green channel film dose. Note the disagreement between planned and measured dose in the shoulder of the high‐dose region. Gamma analysis using 1 mm dose‐to‐agreement criteria is insensitive to such discrepancy in the dose distribution, while Gamma analysis with tighter distance‐to‐agreement criteria (e.g., 0.3 or 0.5 mm) shows failing points in the shoulder of the high‐dose region that match observed dose distribution discrepancies. The Gamma analysis criteria used for this study: 3%, 0.3 mm.

The film measurements were compared to the calculated dose planes using Gamma analysis.^29^ Typical Gamma analysis for patient‐specific IMRT QA may use distance‐to‐agreement criteria of 2–3 mm. However, the measurement scale (percentage of measurement points with passing Gamma values) is often saturated if typical Gamma criteria are used. To determine the appropriate Gamma analysis criteria for this study, the Gamma analysis passing rate results for two representative cases were logged for a variety of dose difference and distance‐to‐agreement criteria and compared to qualitative visual dose profile analysis. The best agreement between passing rate result and visual profile analysis was found for the following Gamma criteria: 3% dose difference and 0.3 mm distance‐to‐agreement. It should be noted that these criteria are likely too strict for planning with conventional target sizes. But, for very small targets such as those found in intracranial SRS planning, a distance‐to‐agreement criteria of 1 mm is quite large relative to the lesion radius (for example, the radius of a 0.5 cc spherical lesion is approximately 5 mm). See Fig. 1 for comparison of the Gamma criteria for one of the representative cases. The value for dose threshold was set to 20% of the maximum film plane dose, which corresponds to roughly 25% of the prescription dose for these cases.

### 2.D. Statistics

Gamma analysis passing rate results for each source size setting were compared to passing rate results for vendor‐recommended source size setting using Student's *t*‐test, assuming two‐tailed distribution with *P* < 0.05 significant.

## 3 Results

### 3.A. Film dosimetry results – gamma analysis

**Figure 2:**
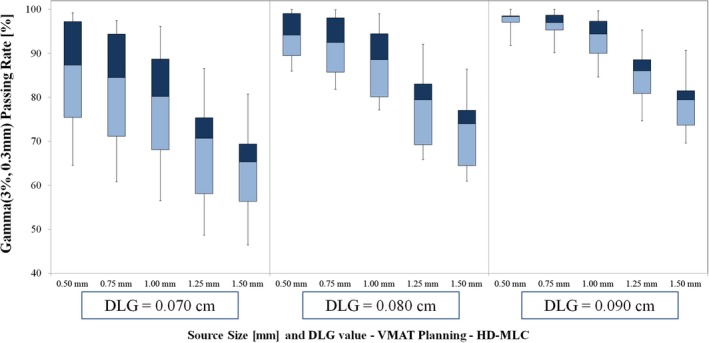
Gamma passing rate results for VMAT planning with Edge linac (HD‐MLC). Note that the highest passing rates occur for the 0.090 cm DLG setting, and the optimal source size varies with DLG setting. For DLG value of 0.090 cm, the 0.50 mm source size results in the highest passing rate (mean ± SD passing rate of 97.51 ± 2.38%). The vendor‐recommended source size setting (1.00 mm) with the same DLG value (0.090 cm) yields a lower mean passing rate result with larger variation–mean ± SD passing rate 93.72 ± 4.96%.

**Figure 3:**
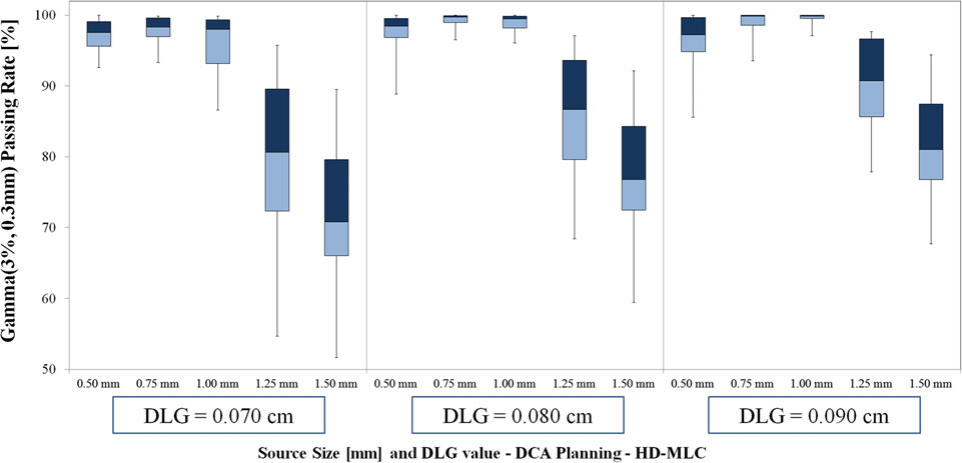
Gamma passing rate results for DCA planning with Edge linac (HD‐MLC). The optimal source size for these data is 1.00 mm for DLG value 0.090 cm (corresponding to the optimal DLG value for VMAT planning). For DLG values of 0.070 cm and 0.080 cm, the highest passing rates occurred for calculations with 0.75 mm source size setting.

The Gamma analysis passing rate results for Edge linac are shown in Fig. 2 (VMAT Planning) and Fig. 3 (DCA Planning). For each plot, the whiskers indicate the maximum and minimum passing rates for each source size setting and DLG value. The first, second, and third quartile values for each combination of settings are also displayed on the plot. Of the DLG settings tested for VMAT delivery with Edge linac, the best overall agreement between measured and planned dose occurred for DLG value of 0.090 cm. For 0.090 cm DLG value, the 0.50 mm source size setting yielded the highest passing rate (mean ± SD): 97.51 ± 2.38% (*P =* 0.01). The passing rates (mean ± SD) for the other source sizes were as follows: 96.25 ± 3.51% (0.75 mm, *P <* 0.01), 93.72 ± 4.96% (1.00 mm), 84.97 ± 6.93% (1.25 mm, *P <* 0.001), and 78.83 ± 7.10% (1.50 mm, *P <* 0.001).

For DCA planning with DLG value of 0.090 cm, the highest passing rate occurred for vendor‐recommended source size of 1.00 mm (mean ± SD): 99.41 ± 0.99%. We note a sharper decline in passing rate for larger‐than‐optimal source sizes (1.25 mm and 1.50 mm) than for smaller‐than‐optimal source sizes (0.50 mm and 0.75 mm) as shown in Fig. 2 and exhibited by the passing rate results (mean ± SD): 96.36 ± 4.41% (0.50 mm, *P =* 0.04), 98.71 ± 2.21% (0.75 mm, *P =* 0.17), 89.96 ± 7.17% (1.25 mm, *P* < 0.01), 81.79 ± 8.30% (1.50 mm, *P <* 0.001).

**Figure 4:**
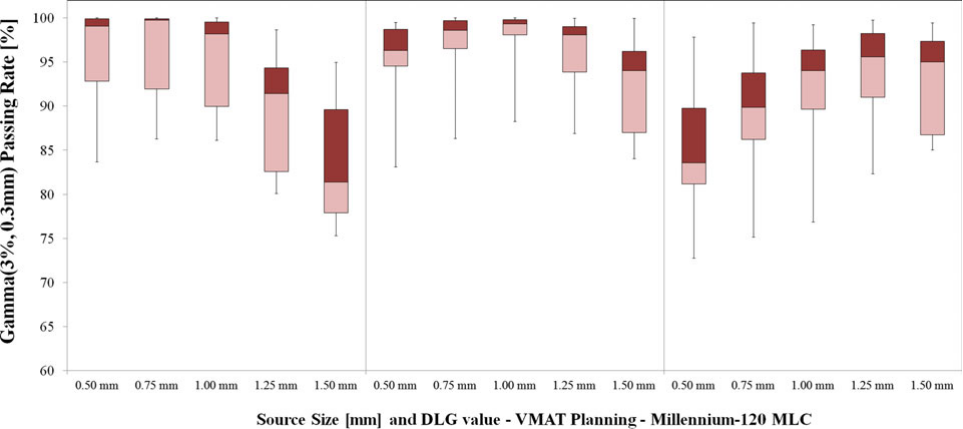
Gamma passing rate results for VMAT planning with TrueBeam linac (Millennium‐120 MLC). Note that the highest passing rates occur for the 0.180 cm DLG setting, and the optimal source size varies with DLG setting. For DLG value of 0.180 cm, the 1.00 mm source size yields the highest passing rates (mean ± SD): 97.84 ± 3.66%. For lower DLG value (0.160 cm), the average passing rate results for 0.50 mm, 0.75 mm and 1.00 mm settings were within 0.7% of one another. The highest passing rate for DLG value 0.160 cm occurred for smaller source size – 0.75 mm setting with mean±SD passing rate of 96.16 ± 5.83%. For the larger DLG value (0.200 cm), the highest average passing rate occurred for larger source size – 1.25 mm setting with mean ± SD passing rate of 93.65 ± 6.19%.

**Figure 5:**
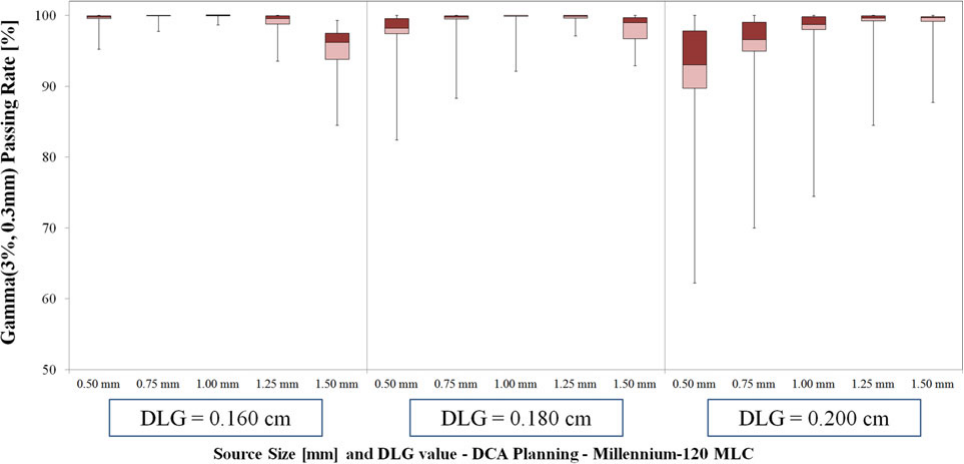
Gamma passing rate results for DCA planning with TrueBeam linac (Millennium‐120 MLC). For 0.180 cm DLG value, the highest passing rates occurred for source sizes of 1.00 mm (99.16 ± 2.47%) and 1.25 mm (99.45 ± 0.99%).

The Gamma analysis passing rate results for TrueBeam linac are shown in Fig. 4 (VMAT Planning) and Fig. 5 (DCA Planning). Of the DLG settings tested for VMAT delivery with TrueBeam linac, the best agreement between measured and planned dose occurred for DLG value of 0.180 cm. For this DLG value, the 1.00 mm source size setting yielded the highest average passing rate (mean ± SD): 97.98 ± 3.06%. The passing rate for the remaining source sizes with 0.180 cm DLG value were as follows: 95.02 ± 4.68% (0.50 mm, *P* < 0.01), 97.22 ± 3.76% (0.75 mm, *P* = 0.066), 95.91 ± 4.33% (1.25 mm, *P* = 0.067), 92.46 ± 5.88% (1.50 mm, *P* < 0.01).

For DCA planning with TrueBeam linac and DLG value of 0.180 cm, the highest mean passing rate occurred for source sizes of 1.00 mm (99.16 ± 2.47%) and 1.25 mm (99.45 ± 0.99%), with no statistical significance in the difference in passing rate for these two source sizes (*P* = 0.62). For all but one TrueBeam DCA case, the 1.00 mm and 1.25 mm source size dose calculations yielded passing rates in excess of 99.50%. The mean ± SD passing rate for the remaining source sizes were as follows: 96.95 ± 5.23% (0.50 mm, *P* = 0.04), 98.62 ± 3.63% (0.75 mm, *P* = 0.17), and 97.94 ± 2.35% (1.50 mm, *P* = 0.26).

### 3.B. Film dosimetry results – dose profile comparison

**Figure 6:**
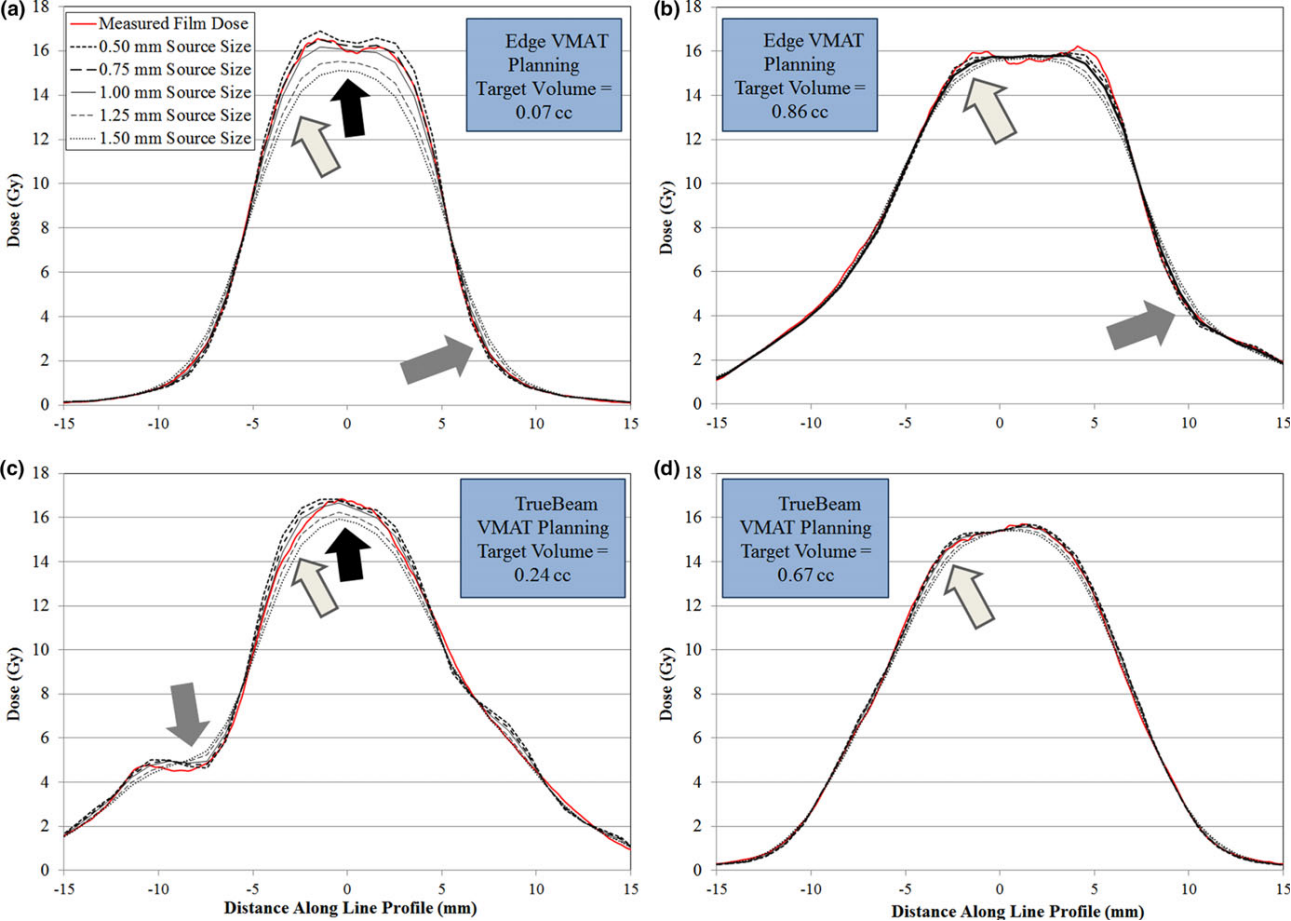
Comparison of measured dose (red solid line) with calculated dose for the source size settings tested for VMAT planning with Edge and TrueBeam linacs. All calculations performed using optimal DLG value: 0.090 cm for Edge linac and 0.180 cm for TrueBeam linac. a, VMAT planning for a small volume target (0.07 cc) with Edge linac. As the source size increases, the magnitude of the central high‐dose region is dramatically reduced (indicated by black arrow), the shoulder of the high‐dose region exhibits blurring (light gray arrow), and the low dose region also exhibits differences as a function of source size (gray arrow). b, VMAT planning for a typical volume target (0.86 cc) with Edge linac. Note the similar blurring of the shoulder of the high‐dose region (black arrow), but no difference in the magnitude of the central high‐dose region. c, VMAT planning for a smaller volume target (0.24 cc) for TrueBeam linac. Note the similar behavior in the central region, shoulder of the high‐dose region, and low dose region to profile comparison in (a). d, VMAT planning for a typical volume target (0.67 cc). Again, there remains characteristic blurring of the shoulder of the high‐dose region without variation in the magnitude of the central high‐dose region.

To highlight the variations in dose calculation with source size setting, line profile comparisons were also generated for several representative VMAT cases for Edge and TrueBeam linacs. A comparison of measured and calculated dose for multiple VMAT plans (one smaller target size and one typical intracranial SRS target size) for Edge and TrueBeam linacs is shown in Fig. 6. For Edge linac and smaller target volume (0.07 cc) shown in Fig. 6 (a) we note two main differences in the high‐dose region among the source size calculations. First, there is an increase in the blurring of the shoulder of the high‐dose region as the source size is increased. In addition, there is a reduction in the magnitude of the central high‐dose region as the source size is increased. For this case, the 0.75 mm source size calculation yields the best agreement with the measured dose. In the low dose region (< 40% of peak dose) for this case, there is also some difference in dose calculation among the calculations with various source sizes. Similar behavior within the high‐dose region for VMAT planning with TrueBeam linac and smaller target volume (0.24 cc) is shown in Fig. 6 (c). For this case, the 1.00 mm source size calculation gives the best agreement with measurement. For more typical intracranial lesions with target volumes in the range of 0.5–1.0 cc (shown in Figs. 6 (b) and 6 (d)), we note the same blurring of the shoulder of the high‐dose region, without the dramatic reduction in the magnitude of the central high‐dose region. For the larger volume case shown in Fig. 6 (b) for Edge linac, the 0.50 mm source size setting yields the best visual agreement between measured and calculated line profiles. For both representative cases shown in Figs. 6 (c) and 6 (d) for TrueBeam linac, the 1.00 mm source size setting yields the best visual agreement between measured and calculated line profiles.

## 4 Discussion

The modern linac, with capabilities for online image guidance and sub‐millimeter end‐to‐end geometric accuracy, has become a popular option for the delivery of stereotactic radiosurgery for treatment of small intracranial lesions. Recently, advances in linac design have included incorporation of stereotactic on‐board imaging systems,^4^, ^5^, ^6^, ^7^ high‐definition MLC,^12^, ^13^ integrated 6 DOF couch,^8^ high‐intensity flattening filter free energy modes,^9^, ^10^, ^11^ and surface imaging systems for tracking.^14^, ^15^ With improvements in the localization and delivery systems, there remains a definite need for accurate modeling of the small field dose delivery within the treatment planning system. The purpose of this study was two‐fold: (1) to present a clinically achievable method to evaluate the source size for small‐field dose calculation, and (2) to use the method to evaluate the ideal source size for flattening‐filter energy modes for two delivery platforms (Varian Edge and TrueBeam machines), MLC leaf models (Millennium120 HD‐MLC and standard Millennium120 MLC), and delivery techniques (DCA and VMAT) used in our clinic.

**Figure 7:**
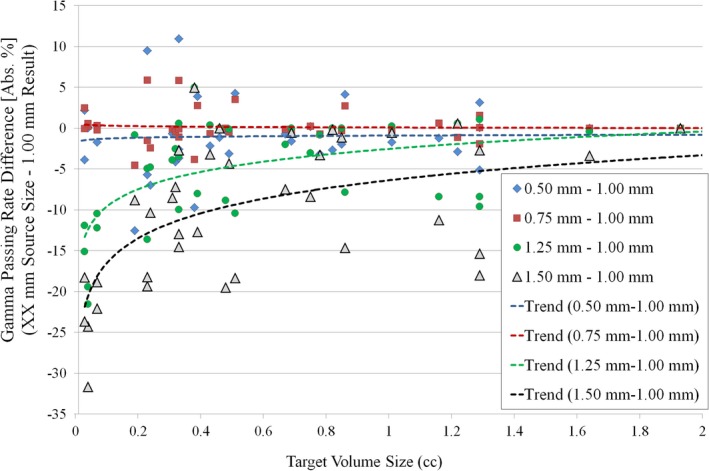
Difference in Gamma Analysis passing rate for each source size tested relative to the vendor‐recommended source size (1.00 mm) for all cases tested (all machine types and all delivery types). Note the sharp decrease in passing rate for smaller target volume size (volume ≤ 0.30 cc) for source sizes larger than the recommended 1.00 mm value (1.25 mm and 1.50 mm).

Previous studies have provided validation of the AcurosXB dose calculation algorithm for a variety of test cases. Vassiliev et al.compared the AcurosXB dose calculation to Monte Carlo for 6 MV and 18 MV beam energy in a heterogeneity slab phantom, finding that AcurosXB and Monte Carlo calculations agreed within 2% (local dose difference) or 1 mm (distance to agreement).^30^ Stathakis et al. compared small field dose calculation within and beyond heterogeneities for several commercially available dose calculation algorithms (including AcurosXB) to Monte Carlo results, finding that AcurosXB agreed within 2% compared to Monte Carlo calculation in lung and bone slab geometry.^31^ Kron et al. evaluated the accuracy of AcurosXB dose calculation for situations with small MLC‐defined segments and larger secondary collimation settings.^24^ Their study included testing the source size (focal spot size) for values of 0 mm, 1 mm, and 2 mm. They found agreement in output factor prediction between AAA and AcurosXB to be within 1% for field sizes ≥ 1 × 1 cm^2^, and found acceptable agreement between planned and measured doses for focal spot size settings of 1 mm or less, DLG value of 1.4 mm, and MLC transmission value of 1.4%. Fogliata et al. evaluated the performance of AAA and AcurosXB for small MLC‐defined open fields and VMAT deliveries.^32^ Their study evaluated 4 VMAT plans in total, but the plan details, including the arc geometry (single partial arc of 140°) and total dose per plan (2 Gy), are not fully representative of typical intra‐cranial SRS VMAT planning at our institution. In addition, the target volume range ([0.3 cc, 7.0 cc]) does not encompass the target volume size that is typically treated at our institution. In particular, our institution tends to utilize multiple arc delivery with typical prescription dosing (on the order of 18 Gy), and the volume of the targets at our institution can be less than 0.10 cc. Our study results indicate that the influence of the source size selection on the dose calculation accuracy is of particular interest for target volume sizes less than approximately 0.30 cc. As shown in Fig. 7, use of source sizes larger than recommended (e.g.*,* 1.25 mm and 1.50 mm in this study) can result in further reduction in Gamma Analysis passing rates for target volume sizes less than approximately 0.30 cc. In addition, the Fogliata et al. study evaluated the algorithm performance with a conventional linear accelerator (Varian Clinac 2100iX) with standard width MLC (Millennium‐120) and standard energy mode (6 MV). In this study, we evaluate various source size settings in AcurosXB for dose calculations of small intra‐cranial targets for VMAT and DCA planning with two linear accelerator delivery platforms utilizing flattening filter‐free energy mode (6 MV‐FFF).

For both AcurosXB and AAA, the Eclipse treatment planning system utilizes a dual‐source dose calculation model, consisting of the primary source and extra‐focal source.^19^ The extra‐focal source is a Gaussian plane source positioned at the edge of the flattening filter distal to the target. For flattening‐filter free beams, such as the 6 MV‐FFF energy mode evaluated in this study, the extra‐focal source modeling is disabled, since the principal element contributing to the need for extra‐focal modeling (i.e., the flattening filter) is not present. The primary source can be tuned using the effective target spot size parameter (entered separately for X‐ and Y‐ directions in the jaw coordinate system). The finite size of the primary source is modeled via Gaussian smoothing of the primary energy fluence, with the spot size parameter representing the FWHM of the Gaussian filter in the isocenter plane. The spot size parameter is coupled with the beam‐limiting device (i.e., jaw or MLC). If the vendor‐recommended spot size parameter (1.00 mm in X‐ and Y‐directions) is used for AcurosXB beam modeling, then the actual source size employed during dose calculation depends on the beam‐limiting device: (1) if the field is delimited by jaws alone, then 1.00 mm is used for X‐ and Y‐directions, and (2) if MLC are used, then the following are used: 1.50 mm (X‐direction) and 0 mm (Y‐direction).^19^ In the current study, we note the difference in behavior for the two MLC models tested. For Millennium‐120 MLC with 0.180 cm DLG value, the vendor‐recommended 1.00 mm source size was suitable for both VMAT planning DCA planning. For Millennium‐120 HD‐MLC, the 0.50 mm source size setting yielded the highest passing rate results for VMAT planning, while the was 1.00 mm source size yielded the highest passing rate results for DCA planning. The differences in these results highlight the need to perform machine‐ and treatment intent‐specific testing, particularly for small‐field delivery using VMAT. When selecting the optimal source size value for a clinical delivery system, it is essential to understand the downside of using smaller source size settings for AcurosXB (relative to the nominal recommended source size). We note that our study findings indicate no statistically significant difference in calculation time among the source sizes tested. However, choosing a source size setting that is too small could lead to overestimation of the size of the shoulder of the high‐dose region. Thus, since the prescription isodose line is often in the shoulder of the high‐dose region, this could lead to an overestimation of the prescription isodose cloud in the planning system. If a tradeoff is needed during commissioning and beam modeling, the user should determine whether underestimating or overestimating the dose level of the shoulder of the high‐dose region is preferable. For our clinic, there was significant interest in using one beam model for each machine. Namely, we looked to avoid using a separate beam model for VMAT and DCA deliveries. It is worth noting that there was no statistically significant difference between the gamma analysis passing rates for 0.50 mm and 0.75 mm (*P* = 0.057) source size values for Edge VMAT delivery, and also no statistically significant difference between the gamma analysis passing rates for 0.75 mm and 1.00 mm (*P* = 0.175) source size values for Edge DCA delivery. In addition, qualitative line profile analysis indicated equivalent agreement between planned and measured dose for the 0.75 mm and 1.00 mm source size data for DCA delivery, as well as between the 0.50 mm and 0.75 mm source size data for VMAT delivery. Thus, we evaluated all of our clinical goals, including: (1) avoid under coverage of the tumor due to overestimation of the prescription isodose cloud, (2) if at all possible, use a single beam model for the Edge machine, and (3) use both quantitative gamma analysis and qualitative line profile analysis. Taking all of these factors into account, the 0.75 mm source size was chosen for our institutional Edge beam model, which included use for both VMAT and DCA deliveries. Our study using Gafchromic EBT3 film and solid water slab phantom provides a feasible and effective method for evaluating beam modeling parameters such as source size and DLG during machine commissioning.

The use of gamma analysis for IMRT QA has been the subject of much scrutiny.^33^, ^34^, ^35^, ^36^, ^37^, ^38^ In particular, gamma analysis using traditional criteria for distance‐to‐agreement (on the order of 2–3 mm) and dose difference (on the order of 2–3%) may not be sensitive to clinically meaningful dose errors when per‐beam IMRT analysis is used. We note several considerations regarding the use of gamma analysis in this study. First, gamma analysis provides a binary result for each pixel (i.e., the pixel either passes or fails the test), and the gamma analysis does not discriminate between delivered dose that is higher or lower than the planned dose. For this reason, commissioning of small‐field deliveries should not rely on gamma passing rates alone; rather, the gamma map and line profile analysis should also be used to give a better understanding of the agreement between planned and measured doses. In this study, we present gamma analysis passing rates and line profile analyses of representative cases. Second, all dose distributions analyzed in this study are composite dose distributions. The dose distributions for each arc are summed in the phantom just as they would in a clinical patient. We believe this avoids one major issue with typical IMRT QA, which is the lack of correlation between per‐beam planar measurements and clinically meaningful dose errors. Third, our institutional film analysis program allowed for the use of relatively small distance‐to‐agreement criteria (on the order of 0.3 mm) for these cases; similar distance‐to‐agreement criteria (0.5 mm) was previously used to validate GammaKnife dosimetry.^39^ The details of our institutional practice for SRS/SBRT QA using Gafchromic film have been published^40^; of note, the film analysis procedure includes a rigid registration routine using mutual information as the similarity metric. For each case, the result of the film analysis image registration is reviewed. It should be noted that the use of such small distance‐to‐agreement criteria (0.3 mm) in the gamma analysis was intended for use in this study and is not part of our institutional clinical routine; the use of such small distance‐to‐agreement relies on (1) robust image registration between the planned and measured dose planes, and (2) sufficiently fine resolution for both planned and measured dose planes. For this reason, we used 1 mm isotropic dose grid setting (the lowest allowed in Eclipse TPS), and exported all plans using 0.098 mm pixel size. Though not all gamma analysis criteria are sensitive to meaningful dose errors, we believe we have shown the 3% dose difference and 0.3 mm criteria as employed in this study to be sensitive to meaningful dose errors in cranial SRS deliveries (see Fig. 1). However, we again stress the usefulness of other complementary analysis tools, such as the use of the gamma map and line profile analysis, and caution against the use of gamma passing rates alone in the commissioning or verification process for SRS VMAT and DCA deliveries. The AAPM Task Group 119 introduced the concept of confidence limits for determining appropriate bounds of IMRT QA results.^41^ The formula used for calculating the confidence limit is as follows: Confidence Limit = ∣mean∣ + 1.96σ Applying this formula to the VMAT film dosimetry data for (1) HD‐MLC (DLG = 0.090 cm, Source Size = 0.50 mm) yields a confidence limit of 92.9% for lower bound of passing rate, and (2) for Millennium‐120 MLC (DLG = 0.180 cm, Source Size = 1.00 mm) yields a confidence limit of 90.7% for lower bound of passing rate. These confidence limits are not generally applicable to all clinics, since they are based on a single institutional QA dataset. Rather, these confidence limits can be used as a qualitative reference when performing similar measurements for commissioning of small‐field VMAT deliveries.

**Figure 8:**
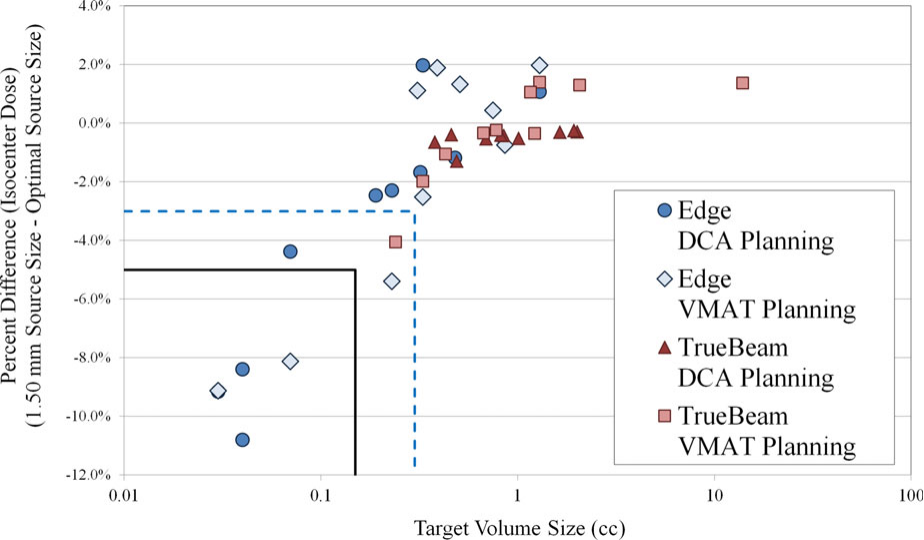
Comparison of isocenter dose for Edge and TrueBeam linacs for VMAT and DCA planning. Dose difference is the isocenter dose 1.50 mm source size relative to isocenter dose for optimal VMAT source size (0.50 mm for Edge linac and 1.00 mm for TrueBeam linac). The blue dashed lines indicate region of target volume less than 0.30 cc and dose difference less than −3%. The black solid lines indicate region with target volume less than 0.15 cc and dose difference less than 5%. The largest dose discrepancy was −10.80% for 0.03 cc volume target.

The dose profile comparison indicated two important regions of dose distribution variation resulting from tuning of the calculation model source size: the shoulder of the high‐dose region and the center of the high‐dose region. First, we note differences in the shoulder of the high‐dose region (Fig. 6). As the source size increases, we note the blurring of the shoulder of the high‐dose region, which occurred for the entire range of target volume sizes tested. This blurring is of particular importance for intracranial SRS planning, since the prescription isodose line is typically in the shoulder region of the dose distribution to allow for sharp dose gradients outside the target volume. Any discrepancy in the full width at 75–85% of the peak dose indicates that the planning system is not correctly calculating the volume of the prescription isodose cloud. In addition, for very small targets (less than approximately 0.30 cc), the central portion of the high‐dose region is also impacted by the source size selection. As shown in Fig. 8, improper selection of the source size in the dose calculation model can result in meaningful reduction in isocenter dose for lesions smaller than approximately 0.30 cc. For very small targets (lesions less than 0.10 cc), we note the presence of discrepancies in isocenter dose up to 10.8%. Of the lesions analyzed in this study with volume less than 0.10 cc, five of the six lesions had dose discrepancies in excess of 8%. These findings reinforce the need to perform dosimetric verification of the dose calculation model at the smallest anticipated target volume size. If the proper source size is not used in the dose calculation model, one potential mitigation strategy would include using a slightly larger margin for intra‐cranial lesions. Line profile analysis in this study indicates that discrepancies between dose calculations with 0.5 mm and 1.5 mm source size setting can routinely approach 1 mm in the shoulder of the high‐dose region. Though an explicit recommendation on lower bound for target size treated for each MLC type is beyond the scope of this work, we have shown that proper tuning of the source size and DLG parameters allow for reliable modeling of the dose delivery with (1) HD‐MLC to target volumes as small as 0.03 cc, and (2) Millennium‐120 MLC to targets volumes as small as 0.24 cc. Other factors, such as the resolution of imaging systems used for planning and localization (e.g., CT, MRI, and CBCT) and the lower bounds of the dose calculation grid size, also play a role in determining the lower bound for target size.

Of note, this study included analysis of conventional and high‐definition MLC models. We note several interesting differences in the passing rate trends for the Edge linac and TrueBeam linac among the entire set of source sizes tested. First, the optimal source size and DLG value resulted in similar mean passing rates for each delivery modality: (1) for VMAT: 97.51 ± 2.38% (Edge) vs 97.84 ± 3.66% (TrueBeam; *P* = 0.81), and (2) for DCA: 99.41 ± 0.99% (Edge) vs 99.16 ± 2.47% (TrueBeam; *P* = 0.77). We believe these similar passing rate results (and corresponding line profile analyses) for the optimal source size and DLG value indicate that the optimal beam model can give similar agreement between planned and measured doses for each MLC type. However, there exists a sharper decrease in passing rate for the Edge data for suboptimal selection of source size and DLG setting. It is worth noting the differences in lesion geometry for the two MLC types. For the Edge linac, there were seven lesions (out of 20 total) below 0.30 cc, with only one such lesion for the TrueBeam linac. In addition, there were nine lesions above 1 cc for the TrueBeam linac, with only two such lesions for the Edge linac. This was intentional, to ensure the lesion volumes were chosen to reflect the intended clinical use of each MLC type. As has been shown in previous studies, the HD‐MLC is better able to deliver stereotactic treatments to small intracranial lesions as compared to standard width MLC.^42^ As shown in Figs. 7 and 8, the dose calculation appears to be most sensitive to variations in source size at the smallest lesion sizes (in this study, we deem the lesion small if the volume is less than approx. 0.30 cc). Under 0.30 cc, variations in source size lead to discrepancies in both the shoulder and central area of the high‐dose region. Thus, though our results indicate no statistically significant difference in the agreement between planned and measured doses in this study for the optimal Edge and TrueBeam beam models, any selection of suboptimal source size (and also DLG value) has the potential to be more impactful, on average, for deliveries involving smaller lesions. In addition, our results indicate a difference in optimal source size for VMAT delivery with these two MLC models, which we attribute to the combination of differences in the MLC leaf end design and the method by which the TPS models the finites size of the source (i.e., a Gaussian convolution blurring). We believe that our study represents the first reporting of the differences in dose calculation as a function of source size with changes in MLC model for Eclipse TPS. In addition, we note the difference in optimal source size as determined using film dosimetry for intensity‐modulated delivery (VMAT) and open‐field delivery (DCA). Some previous studies have characterized the effects of source size using small MLC‐defined open fields with beams at normal incidence and comparing to Monte Carlo calculations, film dosimetry, or other high‐resolution dosimetric data.^17^, ^24^, ^32^ The difference in the film dosimetry results in the current study for VMAT and DCA planning groups (particularly for Edge linac with high‐definition MLC) underscore the need to extend the dose calculation model analysis to include intensity‐modulated deliveries.

The modeling of small‐field deliveries within Eclipse is a combination of the field‐specific output factor (determined from the collimator back scatter factor (CBSF) table in Eclipse), modeling of the MLC leaf end (primarily determined from the DLG value in Eclipse), and the modeling of the source. This study analysis included evaluation of the latter two parameters, but did not fully consider the effects of the CBSF table. However, all beam models were generated using output factor down to jaw sizes of 1 × 1 cm^2^, with small field data measured using a stereotactic field diode. It is important to note that all plans analyzed in this study utilized jaw settings larger than 1 × 1 cm^2^; the smallest jaw setting for this study was 1.6 cm × 1.4 cm (X by Y). Additionally, MLC‐defined small field delivery was validated for field sizes down to 5 mm × 5 mm using multiple detectors. During commissioning, all small field data was measured multiple times and cross‐compared to several detectors for validation, and the calculated output factor data compared favorably with internal Monte Carlo testing. Thus, though we don't explicitly consider the effects of the CBSF table in this study, all beam models were generated with the appropriate selection of detector and the data was validated in multiple ways. This study uses film dosimetry as the primary means to evaluate the accuracy of the dose calculation model. In our clinic, we perform film‐based QA of all intensity‐modulated stereotactic deliveries. In general, the uncertainty in Gafchromic film dosimetry arises from issues with film uniformity, scanner uncertainties, background variations, film handling, and registration between film and calculated dose planes. Our methods, including strict protocols for handling the films, scanning the films in multiple orientations, and keeping scan delay times consistent at 24 hr, minimize the uncertainty in the film result. Through internal testing, we have determined the uncertainty in film absolute dose for small targets to be within 2% at all points of the calibration curve for green channel. Further details on our institutional practice for SRS/SBRT film QA have been published.

## 5 Conclusion

This study highlights the need for tuning of the radiation target source size for the AcurosXB dose calculation algorithm in the context of intracranial SRS dose delivery using DCA and VMAT. In particular, we note the differences in optimal source size values for high‐definition (2.5 mm leaf width) and standard (5 mm leaf width) MLC with flattening‐filter free delivery. Improper selection of the source size can affect the accuracy of the shoulder of the high shoulder for a wide range of intracranial target sizes, and can also have a drastic effect on the magnitude of the central high‐dose region for very small targets (target volume ≤ 0.30 cc).

## Conflict of interest

This work has been supported in part by a grant from Varian Medical Systems, Palo Alto, CA.
